# Supplementary material for: COVID mortality in India: National survey data and health facility deaths
Source: Science. 2022 Jan 6;375(6581):667–71. doi: 10.1126/science.abm5154 (PMC9836201; doi:10.1126/science.abm5154)
Supplement: 20220106-1 [file science.abm5154.v1.pdf]

Cite as: P. Jha *et al.*, *Science*
10.1126/science.abm5154 (2022).

COVID mortality in India: National survey data and health facility deaths

Prabhat Jha[1]*, Yashwant Deshmukh[2], Chinmay Tumbe[3], Wilson Suraweera[1], Aditi Bhowmick[4], Sankalp Sharma[4], Paul Novosad[5], Sze Hang Fu[1], Leslie Newcombe[1], Hellen Gelband[1], Patrick Brown[1]

[1]Centre for Global Health Research, Unity Health Toronto and Dalla Lana School of Public Health, University of Toronto, Toronto, Ontario, Canada. [2]Center For Voting Opinions and Trends in Election Research, Noida, Uttar Pradesh, India. [3]Department of Economics, Indian Institute of Management Ahmedabad, Ahmedabad, Gujarat, India. [4]Development Data Lab, Washington, DC, USA. [5]Department of Economics, Dartmouth College, Hanover, NH, USA.

***Corresponding author. Email: prabhat.jha@utoronto.ca**

India's national COVID death totals remain undetermined. Using an independent nationally representative survey of 0.14 million (M) adults, we compared COVID mortality during the 2020 and 2021 viral waves to expected all-cause mortality. COVID constituted 29% (95%CI 28-31%) of deaths from June 2020-July 2021, corresponding to 3.2M (3.1-3.4) deaths, of which 2.7M (2.6-2.9) occurred in April-July 2021 (when COVID doubled all-cause mortality). A sub-survey of 57,000 adults showed similar temporal increases in mortality with COVID and non-COVID deaths peaking similarly. Two government data sources found that, when compared to pre-pandemic periods, all-cause mortality was 27% (23-32%) higher in 0.2M health facilities and 26% (21-31%) higher in civil registration deaths in ten states; both increases occurred mostly in 2021. The analyses find that India's cumulative COVID deaths by September 2021 were 6-7 times higher than reported officially.

As of January 1, 2022, India reported over 35 million cases of SARS-CoV-2, second only to the United States (US) (*1*). India's official cumulative COVID death count of 0.48 million implies a COVID death rate of approximately 345/million population, about one-seventh of the US death rate (*2*). India's reported COVID death totals are widely believed to be underreports because of incomplete certification of COVID deaths and misattribution to chronic diseases and because most deaths occur in rural areas, often without medical attention (*3*, *4*). Of India's 10 million deaths estimated by the United Nations Population Division (UNPD) in 2020, over three million were not registered and over eight million did not undergo medical certification (fig. S1 and table S1).

Model-based estimates of cumulative COVID deaths through June 2021 in India range from a few hundred thousand to over four million, with most suggesting a substantial official undercount (*5*–*12*) (table S2). However, models start with official reports and apply varying assumptions, leading to wide or implausible estimates. In the absence of near universal and timely death registration and the lack of release of data from India's Sample Registration System (SRS), which tracks deaths in a random sample of about 1% of Indian homes (*13*), alternative approaches are needed to estimate COVID deaths. Recorded increases in all-cause mortality during peak pandemic transmission are likely nearly all caused by COVID infection (*14*). The World Health Organization (WHO) has recognized such counts as a crude but useful method to track the pandemic (*15*). Reports by journalists and NGOs using civil registration system (CRS) data have documented a large increase in deaths from all causes compared with previous years (*16*). Unfortunately, CRS data are reliably available only in states that cover about half of the estimated total deaths in India and may be affected by changes in the level of registration. Given the marked heterogeneity in the temporal patterns of confirmed COVID mortality cases and deaths across states (*17*), and the variable background of mortality rates from chronic diseases affected by COVID infection (*3*), extrapolating from selected states has its limitations.

To fill the gaps in national level estimates, we quantified COVID mortality in India using one independent and two government data sources. The first study is mortality reported in a nationally representative telephone survey conducted by CVoter, an established, independent, private polling agency, which launched the survey on a non-profit basis to help track the pandemic (see Methods, p2 (*18*)). The COVID Tracker survey covers 0.14 million adults (including a sub-study of 57,000 people in 13,500 households with more exact reporting of COVID and non-COVID deaths in immediate family members) (*18*, *19*). In addition, we studied the Government of India's administrative data on national facility-based deaths and CRS deaths in ten states (fig. S2).

The CVoter Tracker survey is a nationally representative, random probability-based computer-assisted telephone interview survey carried out daily to track governance, media and other socioeconomic indicators (*19*). In March 2020, it

began to capture COVID symptoms among adults aged 18 years or older, covering ~2100 randomly selected respondents weekly, drawn from ~4000 local electoral areas in the whole of the country, providing a rolling 7-day average of COVID symptoms and deaths. The survey covers >98% of Indian population by geography, with interviews in 11 languages. The response rate was 55%; 137,289 respondents in all states and union territories were interviewed from March 2020 to July 2021.

Our numerator was defined as the average weekly percentages of surveyed households reporting a COVID death (defined by the household, as medical certification remains uncommon in India, fig. S1). We excluded the 16% of reported COVID deaths that were below age 35 years (confirmed COVID deaths below this age are infrequent; fig. S3) and subtracted a fixed percentage of 0.59% which was an assumed value for reported deaths that did not occur among immediate family members. The assumed value drew on observed background rates during February-March 2021 when few COVID cases or deaths were reported in the official government data (see Methods, p3). Results using survey weights or raw proportions were similar, so we used the latter. We compared these survey-reported COVID deaths to a denominator defined as the expected weekly percentage for all-cause deaths, based on 2020 death totals from the UNPD's comprehensive demographic estimates that combine censuses, survey data and models (*20*) (Fig. 1). India had about 296 million households in 2020 with an average household size of 4.6 (*21*). Dividing this into the 10.16 million deaths estimated by the UNPD in India in 2020 yields approximately 3.4% of households expected to report a death from any cause in that year (with nearly identical results for 2021). To this expected all-cause proportion, we applied the weekly variation observed in the Million Death Study, a large and representative mortality study conducted within the SRS (*3*).

For most of the weeks from June 2020 to March 2021, zero to 0.7% of households in the CVoter survey reported a COVID death. Even the upper value of 0.7% during some weeks corresponded to 20% of the expected annual all-cause death proportion of 3.4%. During the first viral peak, 1.2% of households reported a COVID death (or about 35% of expected all-cause deaths) over ten days from September 24 to October 4, 2020. There was a second sharp increase in reported COVID deaths from mid-April to the end of June 2021, reaching weekly peaks close to 6% of households. From April 1-July 1, 2021, the proportion of households reporting COVID deaths was 3.7%, which was 108% (95% LL and UL, 103-113%) of the expected all-cause deaths of 3.4% (Table 1). The same comparison for June 1-Dec 31, 2020 showed COVID deaths were 8.1% (7.7-8.5%) of expected all-cause deaths.

Applying these proportions to expected overall deaths from June 1, 2020 to July 1, 2021, yielded an estimate of 3.2 million (3.1-3.4) COVID deaths, or 29% (28-31%) of expected all-cause deaths during the 13-month period, including during the interspersed weeks of assumed lower transmission. The majority of COVID deaths India experienced throughout the pandemic occurred from April 1 to July 1, 2021 (2.7 million; 2.6-2.9). Given that the subtraction value for non-household reporting of COVID deaths was somewhat subjective, we ran sensitivity analyses of 50% and 150% of our baseline of 0.59%, yielding estimates ranging from 2.5 (2.4-2.6) to 4.0 (3.8-4.1) million COVID deaths.

The COVID Tracker survey's introductory question focused on flu-like symptoms among immediate family members, but the COVID question asked: "Has anyone in your family or surroundings been infected from Corona Virus?" If the self-reported answer was yes, respondents were asked whether the infected individual died. To address a possible limitation of over-reporting (i.e., COVID deaths in "surroundings" but not in the household), from June 15-Sept 1, 2021 we implemented a sub-study among a randomly selected 10% of households from the COVID Tracker Panel.

We ascertained from approximately 57,000 people in 13,500 households who lived in the immediate household as of January 1, 2019, who died and when, and if the respondent thought the death was due to COVID or a non-COVID cause (Fig. 2 and table S3). The criterion of "immediate household" included parents and unmarried adults. This sub-study recorded 415, 618 and 1074 all-cause deaths in 2019, 2020 and 2021, respectively, corresponding to crude death rates per 1000 people of 7.2, 10.8 and 18.8, respectively (the 2019 crude death rate was similar to the UN all-cause death rate of 8.1/1000 (*20*)). Total COVID deaths reported in 2020 (162) and 2021 (553) corresponded to 1.2% and 4.1% of households reporting a COVID death, comparable to the proportions in the main COVID Tracker survey. The crude death rate in the sub-study more than doubled in 2021 compared to 2019, also consistent with the increase in COVID deaths in the main survey. Compared to 2019, the increase in non-COVID deaths reported during Sept-Oct 2020 exceeded reported COVID deaths but the reverse was true during April-June 2021. This likely reflects the misclassification of non-COVID deaths; COVID infection raises death rates not just from respiratory disease but from vascular disease, kidney disease and other causes (*22*). The Government of India's daily confirmed COVID death totals from June 1, 2020 to July 1, 2021 strongly correlated with the daily death totals in the CVoter main survey (correlation 0.88, p<0.0001). The government's confirmed COVID death totals for each month from April 1, 2020 to July 1, 2021 correlated with the monthly COVID deaths in the CVoter sub-study (correlation 0.84 p<0.001; fig. S4).

We examined two government reported data sources as comparisons to the independent CVoter survey. The first data source comprised facility-based all-cause mortality covering

a non-representative sample of 0.2 million public hospitals and smaller facilities nationally, more than 90% of them rural (*23*) (Fig. 3). Compared to 2018-19, all-cause deaths increased 27% (23%-32%) during July 1, 2020 to May 31, 2021, equivalent to an excess of 0.63 million deaths (0.53-0.73) of 2.32 million expected for the 11 months (Table 1). Much of this excess occurred in April-May 2021 (0.45 million or 71%), reaching a 120% increase over earlier year totals. The increase in facility deaths in the first viral wave was predominantly urban, but deaths in the second wave affected both urban and rural facilities (fig. S5). Compared to 2018-19 totals, the increase in all-cause deaths in April-May 2021 varied across states, with Gujarat reporting a 230% increase and Kerala a 37% increase. In Andhra Pradesh, which had reasonably high coverage of expected rural deaths in facilities, the major increase during April-May 2021 was for deaths of unknown cause, followed by non-tuberculosis respiratory conditions, heart disease and other chronic disease, with a small decrease in death from injuries (table S4). Analysis of increases in overall mortality may therefore better capture the diverse diseases affected by COVID infection.

The second government data source was all-cause deaths in the CRS for ten states with ten or more months of observations (including the interspersed periods between the two viral waves). In these states, the combined median increase, as a percentage of expected deaths based on UNPD death rates, was 26% (21-31%; Table 1). Total excess all-cause deaths were 1.25 million (1.00-1.49) for the ten states that reported about half of national official COVID deaths (*2*). The median ratio of excess to confirmed COVID deaths ranged from six to seven in the two viral waves for these states (table S5).

Our national estimates of 3.1 to 3.4 million COVID deaths help fill a gap in knowledge from focal or model-based studies (table S2). During the 13 months between June 1, 2020 and July 1, 2021, the proportions of excess deaths from COVID in the national survey (28-31%) were comparable to the proportions from all causes in the national facility data (23-32%) or the CRS data in ten states (21-31%). However, the major uncertainties in these estimates are not the relatively narrow confidence intervals, but the assumptions about the non-pandemic mortality rates (*24*). Despite varying methodologies, each with its own limitations, our three studies and those published earlier (table S2) point to a substantial under-reporting of deaths in India's official numbers. Most find a much larger excess of deaths in the second viral wave than in the first. Indeed, the COVID pandemic likely doubled the total death rate from all conditions in April-June 2021.

The estimates of 3.1 to 3.4 million deaths from the independent COVID Tracker survey represent a national COVID death rate per million population ranging from about 2300 to 2500, or approximately 6- to 7-fold the officially reported rate on Sept 1, 2021 (*1*). This would put India's death rate per million population just below the range reported in Brazil (2800/million) or Colombia (2500/million), where registration of deaths is far more complete (*15*). The actual excess deaths in the facilities may be larger as the Government of India has yet to release these data from June 2021 onward. More definitive quantification of excess mortality can be expected once the Registrar General of India re-launches its SRS (*13*) to cover all deaths occurring in 2020 and 2021. Indeed, the extraordinary COVID death totals we document warrant adding a simple question on the age, sex, and date of any death (regardless of cause) occurring in 2020 or 2021 to the 2022 national census. Concurrently, India must expand and improve its death registration and medical certification system, with timelier reporting (*25*). Uncounted or medically uncertified deaths are not uniform, with larger gaps in the poorest states in central India and larger gaps among women than among men (fig. S1 and table S1).

Both the 2020 and 2021 viral waves were characterized by widespread (and, for 2021, mostly uncontrolled) multigenerational transmission of the virus within households, with high levels of antibodies detected (*17*). India's significantly higher COVID death rate in 2021 compared to the lower than expected death rate in 2020 requires further research. The spread of infection to rural areas in 2021 is one factor, but there might also be differences in the pathogenicity between the original virus (Wuhan) in 2020 and the mix of alpha and delta variants accounting for most of the 2021 viral wave (*26*), or other biological predictors of severe infection which changed between these two waves. Similarly, tracking death rates will be essential to understanding the effects of the Omicron wave currently underway in India, or future viral variants.

The strengths of our study are its nationally representativeness and distributed sampling for the survey, use of three data sources, and robust metrics that document increased deaths versus earlier years or expected demographic totals. Our methods are reproducible over time and avoid the limitations of model-based estimates. We focused on increased mortality only in the short time periods of pandemic peaks and assumed no excess mortality between viral peaks. COVID deaths typically are acute, occurring within weeks of infection, but the full effects of COVID infection on various underlying diseases are unknown. Thus, our results are conservative. As we had to rely on household self-reports, we adjusted for possible over-reporting of deaths, and ensured that denominators of CRS deaths considered the underlying deficiencies in death reporting in India. Nonetheless, we faced several limitations. We compared COVID deaths to expected all-cause mortality in the national survey, and in so doing, we might have underestimated the totals that in part arose from increases in deaths misclassified as non-COVID. The metric of excess mortality has limitations as some causes

— notably road traffic accidents, non-COVID infections or other injuries — may have decreased, particularly during COVID lockdowns (table S4). However, the nationally representative Million Death Study, conducted within the SRS, documented that injuries constituted less than one in ten of all deaths in India from 2004-14 (*3*). By contrast, other causes, including those linked to poor mental health, may have risen, as seen in the US (*22*). There might also be an increase in some deaths from neglected health services, as reflected in reports that maternal mortality rose during the pandemic months (*16*), but these may also represent COVID infection among pregnant women (*27*). Changes in non-COVID causes of death are likely to be small compared with the sharp increases in COVID deaths, particularly during the second viral wave. Household self-reports of deaths likely misclassified various conditions that are in fact COVID-related (*28*). The COVID Tracker survey data might have over-reported COVID deaths, as the questions were not restricted to immediate family members, but the sub-survey, which did not have this limitation, yielded very similar results. Rural facility death reporting may have been biased upward if more people than usual sought care during high transmission months. Delays in death registration or a backlog of deaths corrected suddenly might create a spurious peak of excess deaths. However, in the case of Andhra Pradesh, 98% of deaths registered in May 2021 took place within the previous 30 days, not earlier time periods (*16*).

In sum, our study finds that Indian COVID deaths are substantially greater than estimated from official reports. If our findings are confirmed, this may require substantial upward revision of WHO's estimates of cumulative global COVID mortality, which as of January 1, 2022, stood at 5.4 million (*15*).

REFERENCES AND NOTES

1. "The Coronavirus App" (Scriby, Inc, 2021); https://coronavirus.app/map.
2. COVID-19 India Data Operations Group, "Covid19India.org" (2021); https://www.covid19india.org/.
3. G. R. Menon, L. Singh, P. Sharma, P. Yadav, S. Sharma, S. Kalaskar, H. Singh, S. Adinarayanan, V. Joshua, V. Kulothungan, J. Yadav, L. K. Watson, S. A. Fadel, W. Suraweera, M. V. V. Rao, R. S. Dhaliwal, R. Begum, P. Sati, D. T. Jamison, P. Jha, National Burden Estimates of healthy life lost in India, 2017: An analysis using direct mortality data and indirect disability data. *Lancet Glob. Health* **7**, e1675–e1684 (2019). doi:10.1016/S2214-109X(19)30451-6 Medline
4. Office of the Registrar General, India, "Vital Statistics of India based on the Civil Registration System 2019" (2021); https://censusindia.gov.in/2011-Common/CRS_2019/CRS2019_report.pdf.
5. C. Z. Guilmoto, Estimating the death toll of the Covid-19 pandemic in India. medRxiv 2021.06.29.21257965 [Preprint]. 2 July 2021. https://www.medrxiv.org/content/10.1101/2021.06.29.21257965v1.
6. C. T. Leffler, J. D. Lykins V, E. Yang, Preliminary Analysis of Excess Mortality in India During the Covid-19 Pandemic. medRxiv 2021.08.04.21261604 [Preprint]. 27 September 2021. https://www.medrxiv.org/content/10.1101/2021.08.04.21261604v2.
7. A. Malani, S. Ramachandran, "Using Household Rosters from Survey Data to Estimate All-cause Mortality during COVID in India" (2021); https://www.nber.org/papers/w29192.
8. M. Banaji, A. Gupta, Estimates of pandemic excess mortality in India based on civil registration data. medRxiv 2021.09.30.21264376 [Preprint]. 1 October 2021. https://www.medrxiv.org/content/10.1101/2021.09.30.21264376v1.
9. A. Gupta, M. Banaji, "Lessons from India's all-cause mortality data," *The Hindu* (2021); https://www.thehindu.com/opinion/lead/lessons-from-indias-all-cause-mortality-data/article36007188.ece.
10. A. Anand, J. Sandefur, A. Subramanian, "Three New Estimates of India's All-Cause Excess Mortality during the COVID-19 Pandemic" (2021); https://www.cgdev.org/publication/three-new-estimates-indias-all-cause-excess-mortality-during-covid-19-pandemic.
11. R. J. Acosta *et al*., All-cause excess mortality in the State of Gujarat, India, during the COVID-19 pandemic. medRxiv 2021.08.22.21262432 [Preprint]. 25 August 2021. https://www.medrxiv.org/content/10.1101/2021.08.22.21262432v1.
12. A. Bamezai *et al*., Survey evidence of excess mortality in Bihar in the second COVID-19 surge. SocArXiv [Preprint]. 29 July 2021. https://osf.io/preprints/socarxiv/zxq97/.
13. Office of the Registrar General & Census Commissioner, India, "SRS statistical report 2018" (2020); https://censusindia.gov.in/vital_statistics/SRS_Reports_2018.html.
14. P. E. Brown *et al*., Mortality from COVID in Colombia and Peru: Analyses of Mortality Data and Statistical Forecasts. medRxiv 2020.08.24.20181016 [Preprint]. 16 November 2020. https://www.medrxiv.org/content/10.1101/2020.08.24.20181016v2.
15. World Health Organization, "The true death toll of COVID-19: estimating global excess mortality"; https://www.who.int/data/stories/the-true-death-toll-of-covid-19-estimating-global-excess-mortality.
16. R. S, "Deaths By 'Unknown Causes' On National Health Mission Portal 2X Official Covid Toll," *IndiaSpend* (2021); https://www.indiaspend.com/covid-19/deaths-unknown-causes-national-health-mission-portal-covid-toll-760219.
17. A. Velumani *et al*., SARS-CoV-2 Seroprevalence in 12 Cities of India from July-December 2020. medRxiv 2021.03.19.21253429 [Preprint]. 24 March 2021. https://www.medrxiv.org/content/10.1101/2021.03.19.21253429v1.
18. Team CVoter, "COVID-19 Tracker Surveys in India" (2020); https://cvoterindia.com/wp-content/uploads/2020/Covid_Tracker_Methodology_Note.pdf.
19. Team CVoter, "Cvoter News Services"; https://cvoterindia.com/trackers/.
20. United Nations, Department of Economic and Social Affairs, Population Division, "World Population Prospects 2019: Highlights" (2019); https://population.un.org/wpp/Publications/Files/WPP2019_Highlights.pdf.
21. Population Reference Bureau, "International Indicators: Average Household Size" (2020); https://www.prb.org/international/indicator/hh-size-av/map/country.
22. S. H. Woolf, D. A. Chapman, R. T. Sabo, E. B. Zimmerman, Excess Deaths From COVID-19 and Other Causes in the US, March 1, 2020, to January 2, 2021. *JAMA* **325**, 1786–1789 (2021). doi:10.1001/jama.2021.5199 Medline
23. Ministry of Health and Family Welfare, Government of India, "Health Management Information System" (2021); https://hmis.nhp.gov.in/#!/standardReports.
24. M. R. Nepomuceno *et al*., Sensitivity of excess mortality due to the COVID-19 pandemic to the choice of the mortality index, method, reference period, and the time unit of the death series. medRxiv 2021.07.20.21260869 [Preprint]. 23 July 2021. https://www.medrxiv.org/content/10.1101/2021.07.20.21260869v1.
25. P. Jha, Reliable direct measurement of causes of death in low- and middle-income countries. *BMC Med.* **12**, 19 (2014). doi:10.1186/1741-7015-12-19 Medline
26. C. Pattabiraman, P. Prasad, A. K. George, D. Sreenivas, R. Rasheed, N. V. K. Reddy, A. Desai, R. Vasanthapuram, Importation, circulation, and emergence of variants of SARS-CoV-2 in the South Indian state of Karnataka. *Wellcome Open Res.* **6**, 110 (2021). doi:10.12688/wellcomeopenres.16768.1
27. C. Meh, A. Sharma, U. Ram, S. Fadel, N. Correa, J. W. Snelgrove, P. Shah, R. Begum, M. Shah, T. Hana, S. H. Fu, L. Raveendran, B. Mishra, P. Jha, Trends in maternal mortality in India over two decades in nationally representative surveys. *BJOG* 1471-0528.16888 (2021). doi:10.1111/1471-0528.16888 Medline
28. P. Madahar, H. Wunsch, P. Jha, A. S. Slutsky, D. Brodie, Trends in COVID-19-related in-hospital mortality: Lessons learned from nationwide samples. *Lancet Respir. Med.* **9**, 322–324 (2021). doi:10.1016/S2213-2600(21)00080-1 Medline
29. Centre for Global Health Research, cghr-toronto/Indian_Covid_Mortality: Indian Covid Mortality, Zenodo (2021). doi.org/10.5281/zenodo.5796647.

https://github.com/cghr-toronto/Indian_Covid_Mortality.
30. Reuters Staff, "Table-India's mobile subscriber base rises in Jan; Airtel adds 5.9 mln (March 17)," *Reuters* (2021); https://www.reuters.com/article/india-telecoms-users-idUKL4N2LF2GQ.
31. S. Keeter, "From Telephone to the Web: The Challenge of Mode of Interview Effects in Public Opinion Polls" (Pew Research Center, 2015); https://www.pewresearch.org/methods/2015/05/13/from-telephone-to-the-web-the-challenge-of-mode-of-interview-effects-in-public-opinion-polls/
32. P. Gerland, UN Population Division's Methodology in Preparing Base Population for Projections: Case study for India. *Asian Popul. Stud.* **10**, 274–303 (2014). doi:10.1080/17441730.2014.947059
33. Office of the Registrar General, India, "Report on medical certification of cause of death 2019" (2021); https://censusindia.gov.in/2011-Documents/mccd_Report1/MCCD_Report_2019.pdf.
34. P. Novosad *et al.*, devdatalab/covid: DDL COVID repo, Zenodo (2021). doi.org/10.5281/zenodo.5796813. https://github.com/devdatalab/covid.
35. J. A. Lewnard, A. Mahmud, T. Narayan, B. Wahl, T. S. Selvavinayagam, C. Mohan B, R. Laxminarayan, All-cause mortality during the COVID-19 pandemic in Chennai, India: An observational study. *Lancet Infect. Dis.* S1473-3099(21)00746-5 (2021). doi:10.1016/S1473-3099(21)00746-5 Medline

ACKNOWLEDGMENTS

We thank Rukmini S, Srinivasan Ramani, Vignesh Radhakhrishnan, Anurabh Saikia, Mariyam Alavi, Saurav Das, Pratap Vardhan, and Dhanya Rajendran who were crucial in filing petitions to access the various data sources. No individual level patient data were used in the analyses. Unity Health, St. Michael's Hospital, Toronto provided ethics approval (REB 15-231). The opinions expressed are those of the authors and not necessarily the institutions to which they are affiliated. **Funding:** Canadian Institutes of Health Research; Emergent Ventures. **Author contributions:** PJ conceived the study. YD, WS, CT, AB, SS, SHF contributed to statistical analyses. PJ, HG, PN wrote the first draft and all authors contributed. **Competing interests:** The authors declare no competing interests. YD is the director and founding editor of CVoter. No external funding was received for the CVoter COVID Tracker survey or sub-study. **Data and materials availability:** The data used for the main facility and CRS deaths are in the Supplementary Materials (table S6), the primary CVoter data showing daily results are available at https://cvoterindia.com/trackers, and the sub-study data are in table S3. All input data are available at https://github.com/cghr-toronto/Indian_Covid_Mortality (doi:10.5281/zenodo.5796647) (*29*). This work is licensed under a Creative Commons Attribution 4.0 International (CC BY 4.0) license, which permits unrestricted use, distribution, and reproduction in any medium, provided the original work is properly cited. To view a copy of this license, visit https://creativecommons.org/licenses/by/4.0/. This license does not apply to figures/photos/artwork or other content included in the article that is credited to a third party; obtain authorization from the rights holder before using such material.

SUPPLEMENTARY MATERIALS
science.org/doi/10.1126/science.abm5154
Materials and Methods
Figs. S1 to S5
Tables S1 to S6
References (*30*–*35*)
MDAR Reproducibility Checklist

23 September 2021; resubmitted 18 November 2021
Published online 6 January 2022
10.1126/science.abm5154

Table 1. Summary estimates of excess deaths in India nationally and for states with ten or more months of data (including the interim weeks or months that were not pandemic).

Data source	Reference period	Months	UN-estimated deaths in reference period (000s)	Excess deaths (LL, UL) in 000s*	Excess as percentage of UN estimated deaths; mid (LL, UL)*
Survey-based estimates, national	June 1-Dec 31, 2020	7	5979	486 (461, 510)	8.1 (7.7, 8.5)
	April 1-July 1, 2021	3	2539	2739 (2602, 2876)	107.9 (102.5, 113.3)
	**June 1, 2020-July 1, 2021**	**13**	**10 956**	**3225 (3063, 3386)**	**29.4 (28, 30.9)**
Facility-based deaths, national	July 1-Dec 31, 2020	6	1295	180 (169, 191)	13.9 (13.1, 14.8)
	Apr 1-May 31, 2021	2	375	450 (362, 539)	120.2 (96.5, 143.9)
	**July 1, 2020-May 31, 2021**	**11**	**2301**	**630 (531, 730)**	**27.4 (23.1, 31.7)**
Civil registration system deaths, selected states					
Andhra Pradesh	July 1, 2020-June 30, 2021	12	411	207 (155, 259)	50.3 (37.7, 63)
Madhya Pradesh	July 1, 2020-May 31, 2021	11	656	205 (149, 261)	31.3 (22.8, 39.8)
Haryana	July 1, 2020-May 31, 2021	11	195	61 (51, 71)	31.2 (26.3, 36.2)
Maharashtra	July 1, 2020-May 31, 2021	11	813	244 (211, 278)	45.5 (39.2, 51.8)†
Karnataka	July 1, 2020-May 31, 2021	12	660	175 (152, 198)	26.6 (23.1, 30.1)
Himachal Pradesh	July 1, 2020-May 31, 2021	11	31	8 (6, 10)	25.0 (18.4, 31.6)
Tamil Nadu	July 1, 2020-May 31, 2021	12	619	140 (101, 179)	22.6 (16.2, 28.9)
West Bengal	July 1, 2020-May 31, 2021	11	626	138 (124, 152)	22.0 (19.8, 24.3)
Rajasthan	July 1, 2020-May 31, 2021	11	551	50 (38, 61)	17.7 (13.6, 21.8)†
Kerala	Aug 1, 2020-May 31, 2021	10	238	19 (15, 22)	7.8 (6.4, 9.2)
Sub-totals and medians			**4801**	**1247 (1002, 1491)**	**25.8 (21.3, 30.8)**

Notes: Table S6 provides the input data. *For the independent national survey, these are COVID deaths, and for facility and civil registration deaths, these are all-cause excess deaths. †Out of the annual average of 428,000 CRS deaths for pre-pandemic years of Rajasthan (table S1), only 218,000 deaths (table S6) or 51% were available for our calculations. Similarly, for Maharashtra only 66% of CRS deaths were available. This, we have adjusted the percentages in the last column to the available data. The lower (LL) and upper limits (UL) for facility-based deaths and civil registration deaths and percentages are based on the variation in monthly reporting, and those for the national survey are based on the survey margin error of +/− 5%. See Methods for the calculation formulas used for absolute excess deaths and relative excess mortality for the three data sources.

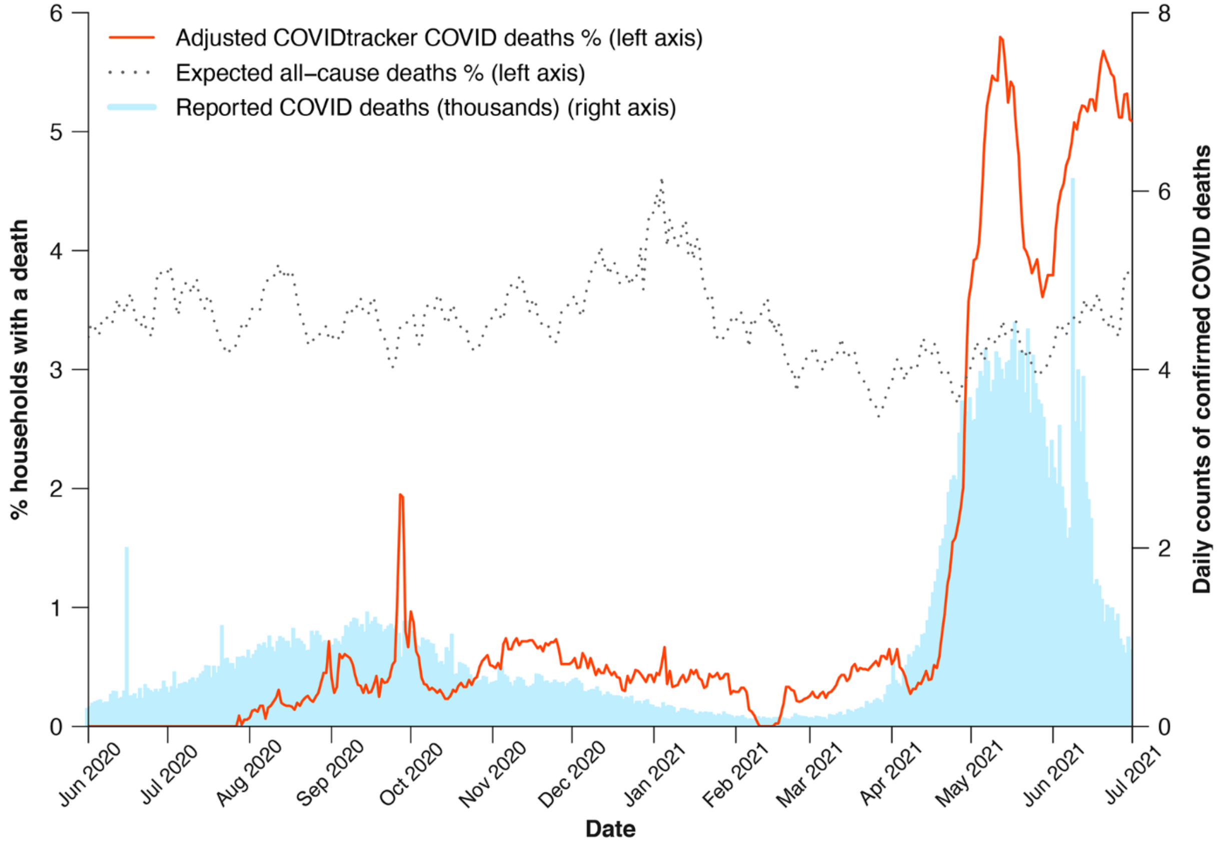

Fig. 1. Percentages of adults reporting daily death in household, expected percentage in 2020, and daily confirmed COVID deaths in India, June 1, 2020-July 1, 2021. COVID Tracker deaths (red line, left vertical scale) represent COVID deaths reported daily (smoothed for rolling 7-day averages) at age 35 or older, less a subtraction value of 0.59% to represent non-household reporting. Expected all-cause deaths (grey line, left vertical scale) per year of 3.4% (see text), with 7-day smoothed weekly adjustment from variation observed among 480,000 deaths in the Million Death Study from 2004-14. Confirmed COVID deaths (blue bars, right vertical scale) are daily reports from Covid19india.org (*2*).

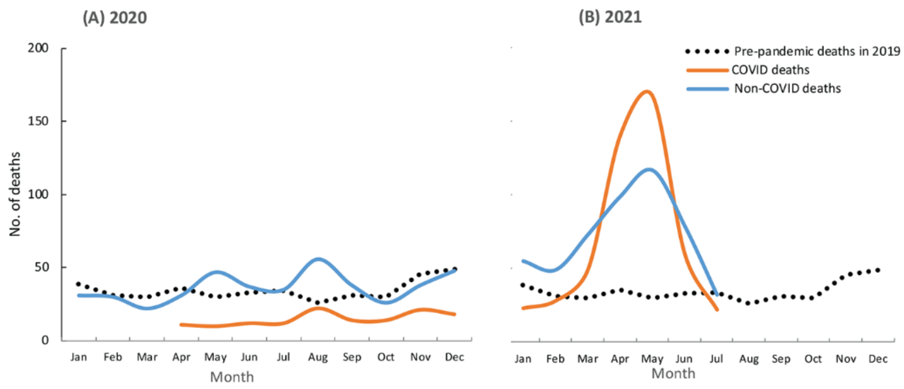

Fig. 2. Monthly reporting of deaths as COVID (including COVID-associated) and non-COVID by month for 2019-2021 in a sub-study of 57,000 adults in 13,500 households within the COVID Tracker survey (*2*). Table S3 provides the input data.

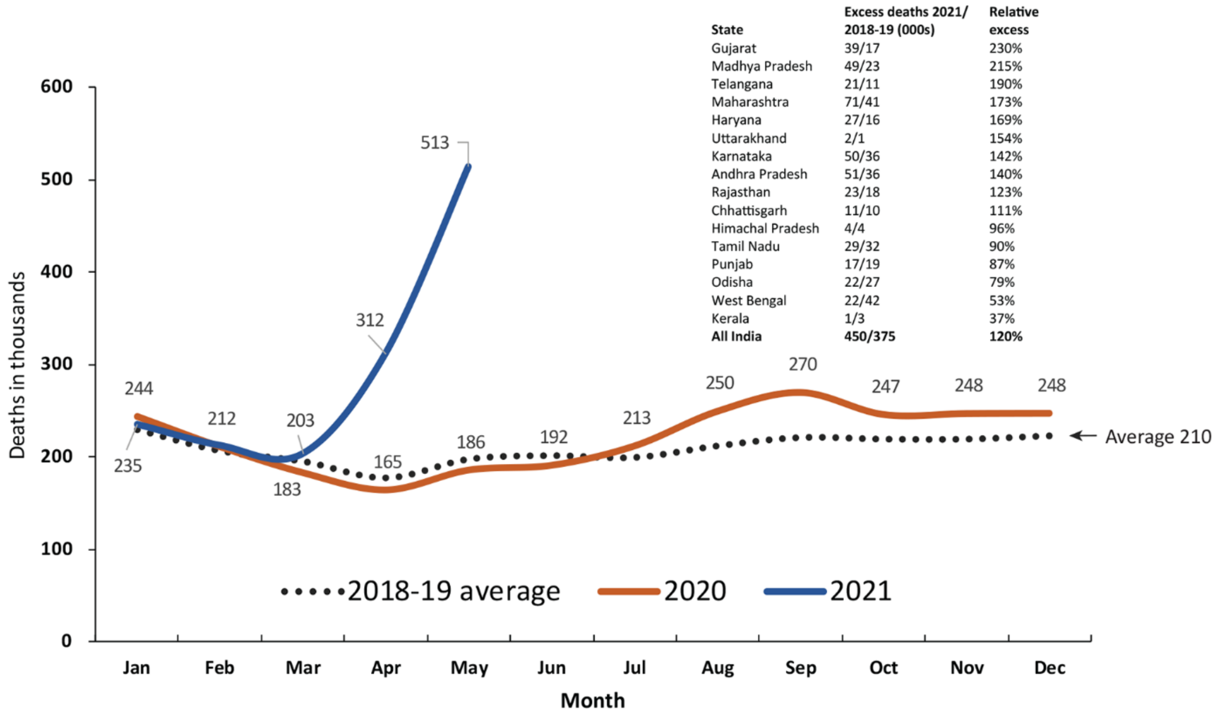

Fig. 3. Reported deaths from all causes in India's Ministry of Health and Family Welfare Management Information System covering 0.2 million health facilities nationally, 2020 and 2021 versus average of 2018-19, by month. The inset shows the increases in selected states and nationally for the April-May 2021 relative to the 2018-19 averages for the same months of comparison. Table S6 provides the input data..
