# Supplementary material for: COVID mortality in India: National survey data and health facility deaths
Source: Science. 2022 Jan 6;375(6581):667–71. doi: 10.1126/science.abm5154 (PMC9836201; doi:10.1126/science.abm5154)
Supplement: Supplementary file 2 — Materials and Methods Figs. S1 to S5 Tables S1 to S6 References (30–35) [file science.abm5154_sm.pdf]

Science AAAS

Supplementary Materials for

COVID mortality in India: National survey data and health facility deaths

Prabhat Jha *et al*.

Corresponding author: Prabhat Jha, prabhat.jha@utoronto.ca

Science **375**, 667 (2022)
DOI: 10.1126/science.abm5154

The PDF file includes:

Materials and Methods
Figs. S1 to S5
Tables S1 to S6
References

Other Supplementary Material for this manuscript includes the following:

MDAR Reproducibility Checklist
Movie S1

Materials and Methods

The three main sources of data and analytic methods are shown in Fig. S2.

CVoter India OmniBus is a nationally representative, computer-assisted telephone interview (CATI) survey carried out daily for political and other socioeconomic purposes (*18, 19*). The methods are published at https://cvoterindia.com/wp-content/uploads/2020/Covid_Tracker_Methodology_Note.pdf. CVoter is an independent polling agency that launched the COVID Tracker survey on a non-profit basis to share key findings openly.

The Indian telecom industry is not required to maintain a "Do not call" registry for unwarranted marketing and sales calls although several operators do so voluntarily. Since opinion research does not seek to market or sell anything; it would not come under these Do not call criteria for the few operators who have voluntary registries.

Cvoter News Services Private Ltd was registered in India as a media and research company in 2000. CVoter has collected data as part of research activities and majority of its research is commissioned and published by mainstream Indian and international media. Media in India are mostly self-regulated. The existing bodies for regulation of media such as the Press Council of India which is a statutory body and the News Broadcasting Standards Authority, a self-regulatory organization, issue standards and Cvoter has been following them (see https://prsindia.org/theprsblog/regulation-of-media-in-india-a-brief-overview). The call centers conducting telephonic surveys are covered under the IT and ITES laws and CVoter had valid IT/ITES license to run the call centers.

CVoter adapted the long-standing public opinion research infrastructure to survey households about their COVID experience. This was done in the national interest with no payments received from any parties. Funding was from existing budgets. Only de-identified data with no individual identifiers are put on our web portal; www.cvoterindia.com for use by others. Since CVoter provided these data for research purpose in the open domain, all academic institutions are free to use it in public interest and do not require any permissions for their non-commercial use.

Consent procedures:

As a part of routine Standard Operating Protocol for the Omnibus operations, consent is taken at first question. If the respondent refuses; it is marked as refusal and the call is terminated. If the respondent is busy at the time of call; then proper appointment is taken for rescheduling of the interview time as per the convenience of the respondent. The consent language is as follows:

> *Hello, I am xyz from CVoter Research Organization. You might have read our surveys in Newspapers or seen TV News programs based on our surveys. We also conduct research for various reputed Universities and academic institutions. Today, I am calling you to conduct a survey on your views on COVID. This survey will take about 15 min of your time. Please be assured that your identity will be safe with us and your answers will only be part of a final analysis on the survey questions.*
>
> *May I conduct this interview?*

At the end of interview consent of respondent is asked again about participation in the CVoter panel. Only those who answer affirmative are empaneled for future interviews. Consent for panel invite:

> *The opinions that you have shared with us are commendable & precise. Would you like to become a part of our panel? We would like to tell you that by Panel, we are implying that we would like to know your opinions on important issues from time to time. It's your priceless*

opinion which can change the country, society & your own life. And we shall become a medium to make your voice heard.

Yes- save number for panel / No- Say thanks and end the survey.

The survey adapted the underlying Omnibus survey to report on COVID symptoms in March 2020, among adults aged 18 years or older, covering about 2100 randomly selected respondents weekly (300 per day in a rolling sample), drawn from nearly 4000 local electoral areas (of which about 1000 were sampled randomly). Random digit dialing stratified for local mobile networks was used to sample India's population, which has 90% or higher coverage with mobile phones or >98% of the Indian population based on geography (*30*).

The visualization of CVoter sampling is shown at https://teamcvoter.com/covidmortalitycoverage. Each dot represents the location of one single respondent. Darker shades mean more respondents on that particular location/state due to more concentrated population. Different colours mark different languages or states in case of same language. The coverage shows robust random probability coverage, as the coverage automatically becomes diverse/heterogeneous demographically when the number of sampling areas (4000) exceeds the number of individuals (2100).

The overall margin of error is +/- 3% at the national level and +/- 5% at the state level, but to be conservative we apply the state level error to the national estimates. All surveys in India generally have relatively lower response rates by females, tribal/indigenous populations and Muslims. We examined data weighted by age, gender, social group, education, voting or not in the last national election, and rural/urban locality using the latest census, the National Sample Survey Organization (NSSO) estimates and past survey information. This yielded a weighted distribution very similar to the Census or NSSO (data not shown). Reassuringly, there was little difference between raw and weighted data, so we use the first for simplicity (data not shown). The survey had higher response rates among men than women who answer the call, but given the focus was on household events, this should not cause any material biases. The response rate in CVoter survey has been consistently high when compared to CATI surveys in the Western democracies (*31*). The main reasons are the high market penetration of mobile phones in India and because CVoter is viewed as a known reliable brand in India.

During the COVID pandemic, the response rate was even higher than during normal operations, due to concerns about the pandemic and the fact that more people were at home during the lockdowns in various parts of India. In the extraordinary circumstances of complete lockdown due to COVID, the survey team decentralized the interview process to 125 staff interviewing from their own homes. This plus the earlier track record of the CATI platform ensured continuity in the polling. The 125 callers conducted calls in 11 languages.

The main survey asked, "*Have you seen flu-like symptoms like high fever, cold, dry cough, or similar symptoms in any family member (within your own household) or in your neighbourhood (people who you normally meet in your day-to-day life)?*" From June 2020, questions were added about COVID infection, hospitalization, or death, and vaccination and testing history. The mortality question asked, "*Has anyone in your family or surroundings been infected from Corona Virus*?" If the answer was yes, respondents were asked whether the infected individual died. From May 15, 2021, the age of death was ascertained.

To ascertain deaths directly in the household and the contribution of COVID or non-COVID deaths, a follow up survey in a randomly selected 13,500 households covering the whole country, with a population of about 57,000 conducted from July 1 to Sept 15, 2021. This more directly ascertained about

deaths, since January 1, 2019 in the immediate family/household as well as the total number of people living at the start of the period. Further questions ascertained if the respondent thought the death for each sex was due to COVID (typically respiratory infections) or causes related to COVID. We asked the cause of death as an open-ended question that the family reported. Independent medical confirmation of these causes, while desirable, was not possible. Deaths were coded to COVID-related cases and where other medical reasons were given, coded as "Covid+". The second category were deaths not directly or indirectly related to COVID. Table S3 provides the results of this sub-survey.

Statistical methods for the main CVoter survey

We made two adjustments to the main CVoter data to calculate the numerator of the proportions of households reporting a COVID death. First, we excluded deaths before age 35, which are unlikely to be from COVID (*2*) (16.6% of reported deaths, thus retaining 83.4% of COVID deaths). This percentage was derived from the survey for May 15-July 1, 2021, and showed a consistent pattern during this period. Thus, we applied this percentage to all COVID deaths throughout the study period from June 1, 2020 onwards. As validation, in the state of Karnataka, confirmed COVID deaths in the second viral wave occurred at younger ages, especially among men (Fig. S3), but in both sexes combined, only 3.4% and 4.9% of confirmed COVID deaths occurred before age 35 years in the first and second viral wave, respectively. In Gujarat, excess deaths also showed a modest downward shift in age for women but not men between 2020 and 2021 (data not shown).

Second, COVID deaths may have been over-reported because the second question described above may not have been restricted to immediate household members. To correct for this, we subtracted for proportions of households that reported a COVID death (also above 35 years or older), but which were assumed to occur outside the household. We based this percentage of 0.535% on the observed average survey prevalences of COVID deaths from February 15, 2021 to March 31, 2021 when confirmed daily COVID deaths on Covid19India.org were reported as below 200 in the whole country (2). We assumed that this number represents the background rate of adults who knew of a COVID death among their wider social circles, but not in their household, and that such over reporting was constant for the study period. We subject this assumption to a sensitivity analysis using 50% (0.292) or 150% (0.877) of the baseline value of 0.535%.

Thus, the calculations were as follows:

(1) *i*= **viral wave 1** (June 1-Dec 31, 2020) or **viral wave 2** (April 1-July 1, 2021)

Since close to 90% of the confirmed COVID deaths reported from April 1 to September 1, 2021 occurred from April 1 to July 1, we assign as zero the value of any deaths after July 1. This is consistent with our approach of adopting conservative estimates of COVID mortality.

(2) **R*i***=proportion of households reporting a COVID death in the CVoter data

R1=1.036% *
R2=5.145% *

(3) **D*i***= (R*i* X *0.834*)-*0.585*
0.834 is the proportion of COVID deaths at 35 years or older as described above.

0.585 is the absolute subtraction value as described above.

D1= (1.036%*0.834)-0.585=0.279%
D2= (5.146%*0.834)-0.585=3.706%

We compared these COVID death proportions to the total deaths for that calendar year (2020 or 2021), assuming no COVID deaths during the non-pandemic peak months. The expected deaths were based on the 2020 United Nations (UN) demographic estimates of total deaths (2021 projected deaths yielded nearly identical results) (*20*). The calculations were as follows:

(4) **H=**Excepted percentage of households reporting any death, regardless of cause in 2020
=UN death totals (10.157 million)/Total households, as reported by the Population Reference Bureau of India (*21*) (295.65 million) =3.435%

(5) **P*i*=**Proportion of households reporting a COVID death compared to overall expected deaths in the reference year
=D*i*/H

P1= 0.279/3.435=8.1%
P2=3.706/3.435=107.9%

(6) **C*i*=**COVID deaths during reference period (in months) for each viral wave
=UN death totals X P*i*

C1=5.979 million X 8.1%= 0.486 million
C2=2.539 million X 107.9%=2.739 million
Sum of C1 and C2= 3.225 million

[*] 95% lower (LL) and upper limits (UL) were derived from the polling error of +/- 5% (state-level, larger than the national polling error) applied to R*i*.

Statistical methods for the sub survey

We compared the COVID and non-COVID deaths recorded per month in 2020 or in 2021 to those in 2019, with no further adjustments. The crude death rate was the number of deaths from all causes in each year divided by the total survey population of 57,256.

Additional analyses: Daily variation in expected deaths throughout a calendar year enables comparison of COVID deaths on specific days (Figure 1). We applied daily variation in all-cause mortality from the Million Death Study data from 2004-14 (*3*) to 2020 deaths. Finally, we applied a correlation statistic to compare the adjusted COVID deaths to confirmed COVID deaths, reported on covid19India.org (*2*), covering official and media reports daily from June 1, 2020 to July 1, 2021 (Fig S4).

Facility-based death reporting

The Ministry of Health and Family Welfare tracks key outcomes relevant to national programs through an online Health Management Information System (HMIS) that covers about 0.2 million public hospitals and smaller facilities nationally, more than 90% of them rural (*23*). Data are updated monthly, including

details on age group and broad cause of death for adolescents and adults combined (age 15 or older). Causes include respiratory diseases including infections (other than tuberculosis), heart disease/hypertension related, other known chronic disease, accidents/burns or causes not known.

During 2018-19, the HMIS reported an average of 2.50 million annual deaths. The Registrar General of India's Sample Registration System, used for continuous monitoring of key demographic data in the country reports that in 2018, 47.8% of all deaths occurred in facilities, which multiplied by the UN death totals for 2020 equals 4.85 million deaths in facilities. Thus, the HMIS captures about 52% of all facility-based deaths or about 25% of all facility and home deaths in the country (*13*).

For the facility-based death reporting (*23*), we used the average monthly counts of all-cause deaths during the peak of the first viral wave (July 1-Dec 31, 2020) (*17*) and the second viral wave (April 1-May 31, 2021, the latest available as data for June have yet to be released). We defined pandemic deaths as Ri, where i represents the first or second viral wave. We compared these to the average all-cause deaths for these same months for 2018-19, defined as Gi. We calculated the increase in deaths using the following formula for the whole country and stratified analyses by rural and urban areas (Fig. S5).

$Relative\ increase\ in\ deaths\ (X)\ = (\sum_i Ri\ /\ \sum_i Gi\ ) - 1$ i=1,2 Pandemic periods

Ri: Pandemic death, Gi: Pre-pandemic death

Civil Registration System (CRS) data

India's 1969 Registration of Births and Deaths Act requires compulsory registration of the facts of births and deaths (but not causes of death). A local registrar for each village in rural areas and for each municipality in urban areas is responsible for reporting, which is through electronic entry. The latest CRS annual report is for 2019 (*4*), but we obtained disaggregated data for 2020 and 2021 for this analysis from journalists and local NGOs, who received data through India's Right to Information Act or through administrative requests. Our sample includes all states and cities where the proportion of registered deaths before 2020 covered at least 50% of expected deaths and which had at least ten months of data (to consider non-pandemic months between the viral waves) yielding ten states (Andhra Pradesh, Haryana, Himachal Pradesh, Karnataka, Kerala, Madhya Pradesh, Maharashtra, Rajasthan, Tamil Nadu, West Bengal). Table S2 provides data for all states, including those not meeting the inclusion criteria as well as for cities and Table S7 provides the input data. We report only aggregate deaths, as sex and socioeconomic-stratified data were incomplete.

We compared CRS deaths in the first viral wave (July 1-Dec 31, 2020 in most places (*17*)) and the second viral wave (April 1-June 30, 2021) to deaths during previous control years (2015-19 or the largest available subset of these years) for the same months. The total excess deaths were the difference between these two comparison periods.

Increases or decreases in registration, as well as delays in the registration process, may affect reported deaths and hence use of registered deaths in denominator in calculation of relative excess death could be erroneous. Thus, the main calculation compared the absolute excess deaths noted above to the estimated baseline of expected deaths in pre-pandemic months. The latter were calculated by partitioning the UN Population Division national death estimates into states by rural/urban status based on the SRS proportions of 2016-18 average deaths (Table S2) (*3, 20*).

The main calculations used for state level relative excess deaths is thus

$$Relative\ excess\ death\ (X) = (\sum_i (Ri - Gi) / \sum_i Ai)\ \text{i=1,2 Pandemic period}$$

Ri: Reported CRS deaths in the pandemic months, Gi: Reported CRS deaths in comparable previous non-pandemic months, Ai: expected deaths based on the UN death rates applied to the state/city.

This yielded more conservative relative excess mortality than using just the CRS baseline years, as the UN-based denominator would consider any surge in reporting of deaths during the pandemic months, one common criticism of the CRS data (*9*). Moreover, the UN demographic estimates adjust for the approximate 15% undercount in deaths in the SRS (*3, 32*). We calculated 95% LL and UL using the variability of monthly facility or CRS reporting during the pandemic months of 2020 and 2021 and the corresponding months in pre-pandemic years.

All statistical analyses were conducted in SAS 9.4 and R 4.0.3.

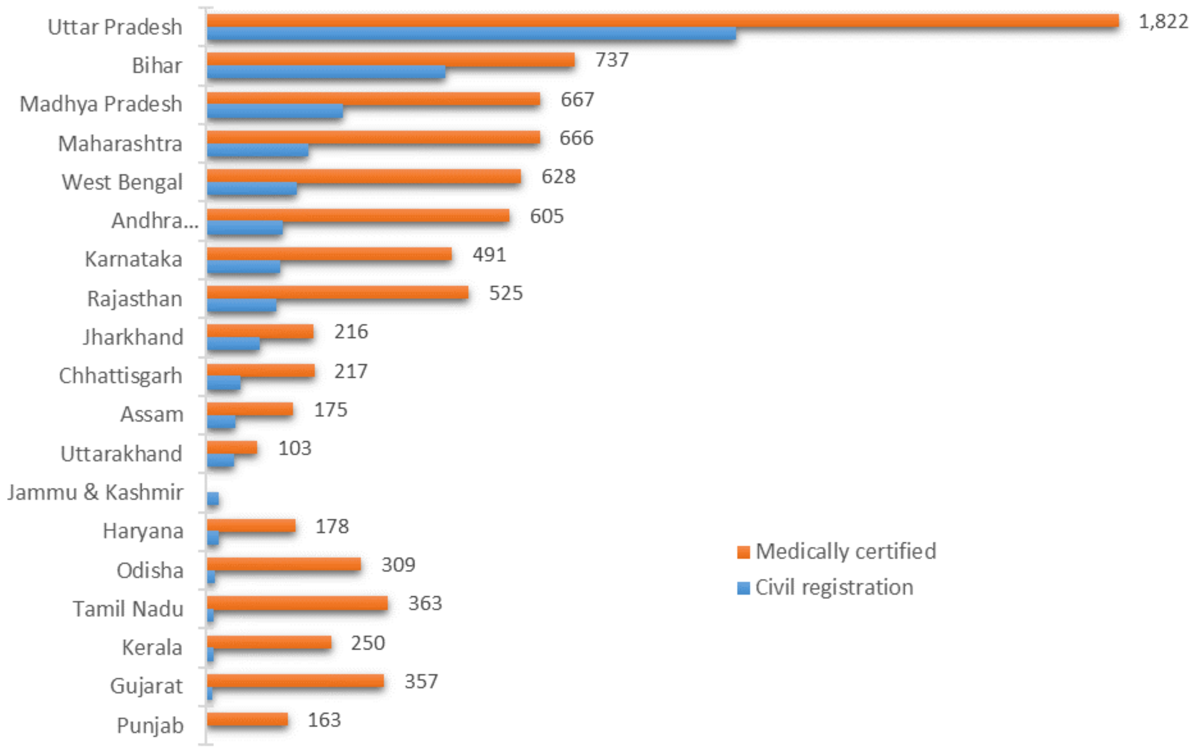

Fig. S1. Uncounted civil registration deaths and uncounted medically certified deaths by major states in India in 2019

Uncounted deaths are UN death totals for India, partitioned to each state using the Sample Registration System less Government of India's registered deaths. Uncounted medically certified deaths subtract those which have undergone medical certification. Overall, of about ten million annual deaths in India, three million deaths are uncounted in death registration and eight million have not undergone medical certification. See Table S5 for details, including data on the larger levels of undercounts among females.

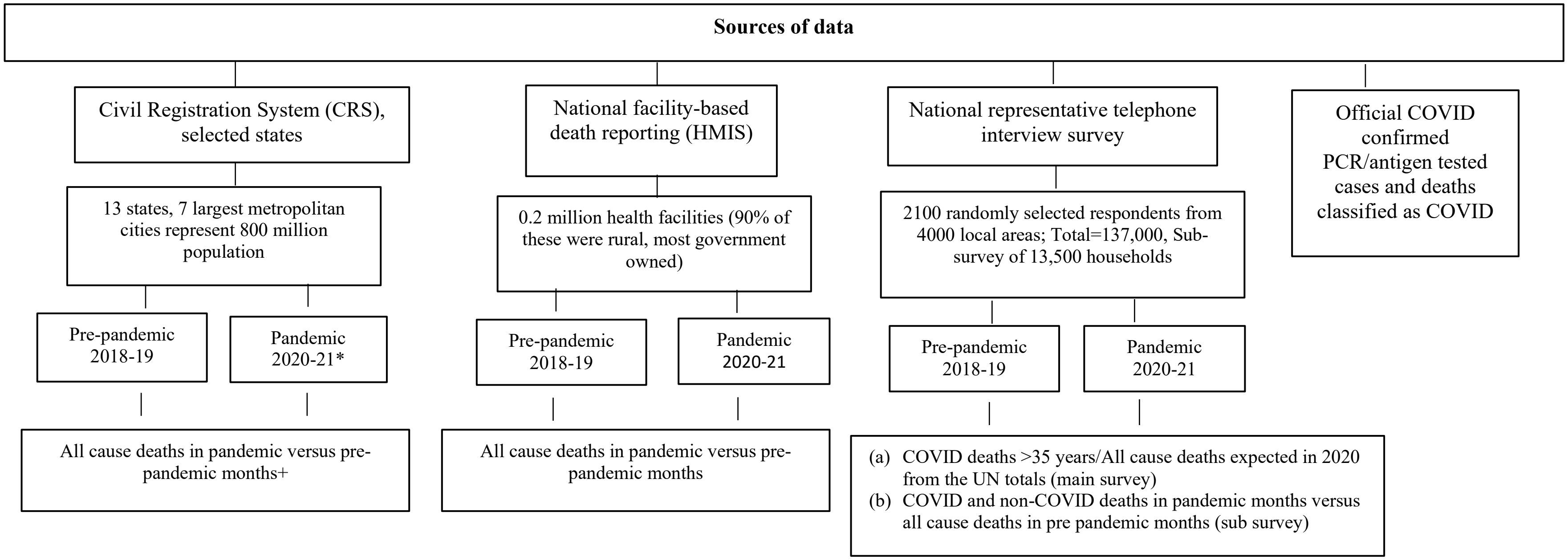

Fig. S2. Flow diagram – data sources, methods, estimation and assessment of excess COVID and all-cause deaths in India

* Pandemic months are variously defined for 2020 and 2021, depending on the viral wave and on available data
+ Deaths in pre-pandemic months use the expected totals based on the UN totals of deaths in that state (see Methods) and not reported deaths in the CRS.

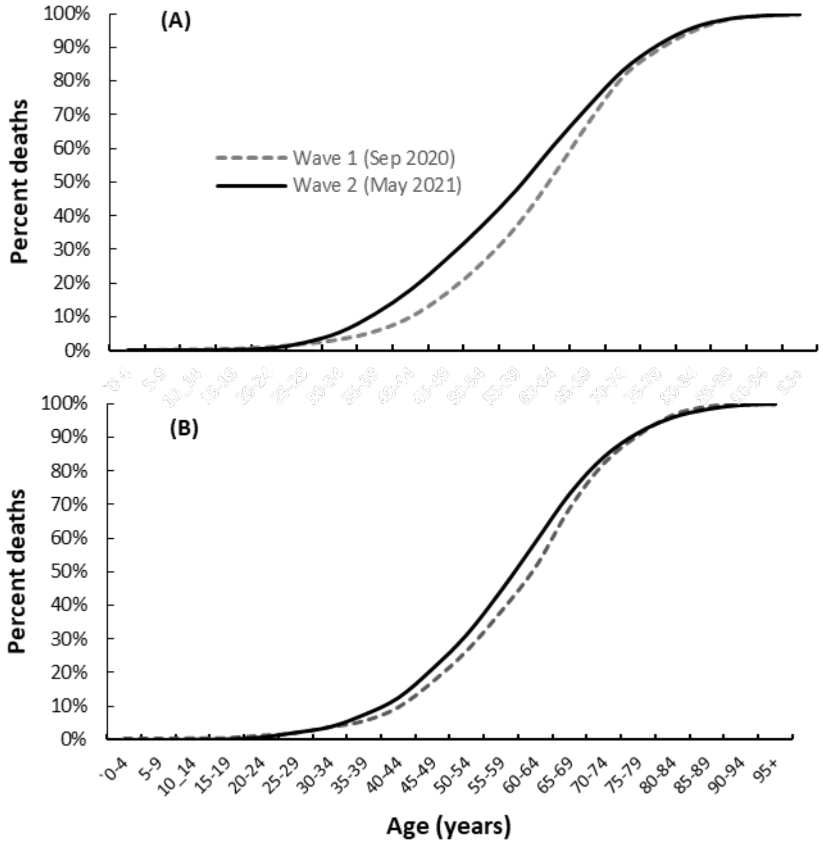

Fig. S3. Cumulative age patterns for confirmed COVID deaths by sex for the first and second waves in Karnataka

(A) Male deaths, (B) Female deaths. We used deaths reported by the Karnataka state government bulletins (3,200 deaths in Sep 2020 in wave 1 and 13,000 deaths in May 2021 in wave 2). Deaths in wave 2 were younger than in wave 1: For both sexes combined, the median ages of death at wave 1 and wave 2 were 64 and 60 years, respectively. For both sexes combined, the proportion of confirmed COVID deaths before age 35 years was 3.4% and 4.9%, respectively in the wave 1 and wave 2.

Source: Ministry of Health, State Government of Karnataka, COVID-19 Media Bulletin (https://covid19.karnataka.gov.in/govt_bulletin/en)

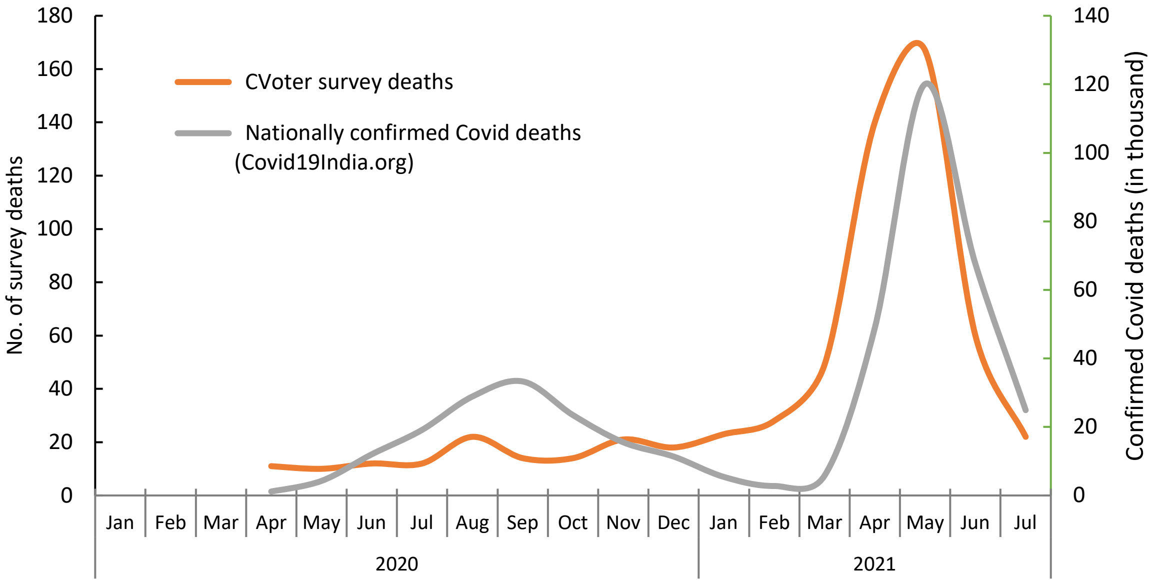

Fig. S4. Comparison of CVoter sub-study of 57,000 people in 13,500 households comparing monthly COVID deaths with national confirmed COVID deaths 2020-21

Notes: Nationally confirmed COVID deaths were extracted from the data portal maintained by Covid19India.org (*2*). Pearson correlation between the COVID deaths of CVoter sub-study and the nationally confirmed COVID deaths from April 1, 2020 to July 1, 2021 was 0.82, $P<0.0001$.

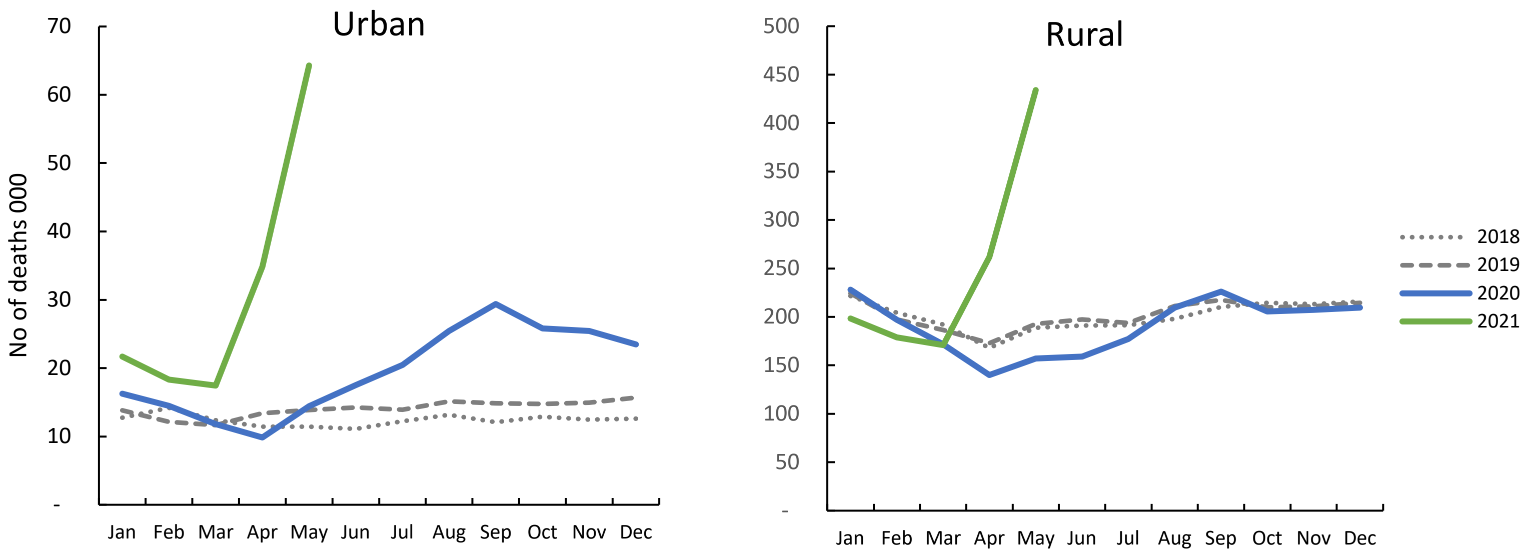

Fig. S5. Reported deaths from all causes in India's Ministry of Health and Family Welfare Health Management Information System by rural and urban status

Notes: We made the following corrections to the published data. First, from the March 2019 data for rural India we subtracted 40,000 due to a typo noted for Warangal district in Andhra Pradesh. From the September 2019 data for urban India, we subtracted 9,000 due to typo noted for Jodhpur district in Rajasthan.

State (Population in millions)	UN estimated deaths (0000) 2019*	CRS deaths 2017-19 (000)			Medically certified deaths 2017-18 (000)	% registered but not medically certified		% Uncounted deaths among		
		Total	Urban	Female		All India	Urban	Both sexes for CRS	Females for CRS #	Both sexes for medical certification
(1)	(2)	(3)	(4)	(5)	(6)	(7)=1-(6)/(3)	(8)=1-(6)/(4)	(9)=1-(3)/(2)	(10)	(11)=1-(6)/(2)
**India (1,380)**	**10,008.6**	**7,018**	**2,983**	**2,846**	**1,355**	**80.7**	**54.6**	**29.9**	**59.3**	**86.5**
Andhra Pradesh /Telangana (91.4)	711.5	558.7	231.2	223.7	106.2	81.0	54.1	21.5	80.5	85.1
Assam (35.3)	207.2	148.9	41.3	59.7	32.1	78.5	22.3	28.2	64.5	84.5
Bihar (123.3)	756.2	278.3	72.3	105.9	19.3	93.1	73.3	63.2	57.6	97.4
Chhattisgarh (30.1)	250.5	180.3	65.6	75.4	33.9	81.2	48.3	28.0	51.9	86.5
Delhi (19.9)	83.1	142.3	125.6	54.6	84.8	40.4	32.5		34.1	
Gujarat (69.5)	441.0	428.0	197.7	172.5	84.4	80.3	57.3	2.9		80.9
Haryana (29.8)	208.9	183.2	82.7	67.6	30.4	83.4	63.2	12.3	85.9	85.4
Jharkhand (38.5)	220.6	112.8	45.1	45.9	5.0	95.5	88.8	48.9	60.4	97.7
Karnataka (69.5)	639.0	491.3	218.8	192.4	147.9	69.9	32.4	23.1	80.5	76.9
Kerala (35.3)	279.3	264.1	98.8	118.4	29.2	88.9	70.4	5.4	73.3	89.5
Madhya Pradesh (84.3)	704.0	429.4	168.8	164.0	37.4	91.3	77.8	39.0	53.0	94.7
Maharashtra (127.1)	873.4	669.6	393.0	282.8	207.3	69.0	47.3	23.3	68.9	76.3
Odisha (46.5)	349.1	331.5	86.8	141.9	39.7	88.0	54.3	5.0	65.6	88.6
Punjab (31.2)	196.9	212.9	100.9	87.8	34.0	84.0	66.3		6.2	82.7
Rajasthan (79.7)	582.0	439.8	145.7	156.2	56.7	87.1	61.1	24.4	55.5	90.3
Tamil Nadu (82.3)	611.9	596.1	300.6	241.7	248.7	58.3	17.3	2.6		59.3
Uttarakhand (11.6)	107.4	49.4	21.1	18.9	4.1	91.6	80.4	54.0	57.5	96.2
Uttar Pradesh (227.5)	1,865.1	807.5	282.6	352.0	43.4	94.6	84.6	56.7	47.6	97.7
West Bengal (102.3)	675.9	495.1	218.5	205.0	47.7	90.4	78.2	26.8	65.8	92.9
Jammu & Kashmir (15.0)	66.9	40.3	14.4	17.6	NA	NA	NA	39.8	31.0	NA

Notes: CRS=Civil Registration System *UN death estimates: SRS death rates (predicted rates from a fitted negative binomial model) applied to United Nations estimated population and then adjusted to UN estimated deaths. We use 2019 UN deaths as these are closest to the CRS published data. Results using 2020 deaths were very similar. # SRS-UN estimated female death not shown in this table.

Sources of data: SRS Vital Statistics reports (*13*), Medical Certification of Cause of Death (*33*), Vital Statistics of India civil registration system (*4*), World Population Prospects of United Nations Population Division (*20*).

Table S1. Civil registration uncounted deaths in major Indian states

Study	Outlet	Excess Mortality Estimates in Millions	Reference Period	Database
Guilmoto (2021) (*5*)	MedRxiv Pre-Print, July 2021	2.2	Till May 2021	Age-Sex COVID Death rates: Kerala, Indian Railways, MLA's
Leffler, Lykins and Yang (2021) (*6*)	MedRxiv Pre-Print, Sep 2021	2.0-3.6	Till Aug 31, 2021	CRS
Malani and Ramachandran (2021) (*7*)	NBER Working Paper, Aug 2021	4.5	Till June 2021	CMIE survey
Banaji and Gupta (2021) (*8*)	MedRxiv Pre-Print, Oct 2021	2.8-5.2	April 2020-June 2021	CRS
Gupta and Banaji (2021) (*9*)	The Hindu Newspaper, Aug 20, 2021	3.5-3.7	April 2020-June 2021	CRS
Anand, Sandefur and Subramanian (2021) (*10*)	CGD Working Paper, July 2021	3.4-4.9	Till June 2021	CMIE survey, CRS, IFR estimates
**Micro-Studies**				
Acosta et al. (2021) (*11*)	MedRxiv Pre-Print, Aug 2021	16,000 for 54 Gujarat Municipalities	March 2020-April 2021	Wall of Grief Database on Gujarat Death Registers
Bamezai et al. (2021) (*12*)	Pre-Print, Aug 2021	0.3 million for Bihar	April-June 2021	Private survey-based extrapolation
Lewnard et al (2021) (*35*)	Lancet Global Health, Dec 2021	26,000 for Chennai	March 2020-June 2021	CRS

Acronyms: CRS= Civil Registration System, HMIS= Health Management Information System, CMIE= Centre for Monitoring Indian Economy, MLA= Member of Legislative Assembly. Other model-based estimates not shown in the above table include Gamio and Glanz (https://www.nytimes.com/interactive/2021/05/25/world/asia/india-covid-death-estimates.html), Institute for Health Metrics and Evaluation (https://covid19.healthdata.org/india), and Purkayastha et al. (https://doi.org/10.1186/s12879-021-06077-9).

Table S2. Previous focal or modelling studies providing estimates of excess or COVID deaths in India

Year	Month	COVID death	COVID-associated death	Non-COVID death	Cause not certain	Total
2019	Jan			28	11	39
	Feb			20	11	31
	Mar			19	11	30
	Apr			20	16	35
	May			20	10	30
	Jun			22	11	33
	Jul			22	12	34
	Aug			15	11	26
	Sep			20	11	31
	Oct			18	12	30
	Nov			34	12	45
	Dec			35	14	47
	**Total**			**273**	**142**	**415**
2020	Jan	-	-	31	2	33
	Feb	-	-	30	4	34
	Mar	-	-	22	6	28
	Apr	9	2	31	2	44
	May	8	2	47	7	64
	Jun	12	-	37	3	52
	Jul	8	4	35	2	49
	Aug	21	1	56	3	81
	Sep	9	5	38	7	59
	Oct	7	7	26	1	41
	Nov	16	5	38	4	63
	Dec	16	2	48	4	70
	**Total**	**106**	**28**	**439**	**45**	**618**
2021	Jan	17	6	55	6	84
	Feb	27	1	49	5	82
	Mar	43	6	73	10	132
	Apr	123	17	99	27	266
	May	147	20	117	21	305
	Jun	49	11	79	12	151
	Jul	19	3	32	-	54
	**Total**	**425**	**64**	**504**	**81**	**1074**
	**Overall total**	**531**	**92**	**1216**	**268**	**2107**

Notes: There were 184 deaths in 2019 (62 non-COVID and 122 cause not certain) without a month assigned. We distributed these deaths to other months in the same proportion as known deaths.

Table S3. CVoter sub-panel of 13,500 households with 57,000 people reporting of COVID deaths, COVID-associated deaths and non-COVID deaths by month from 2019-2021

Cause of death in adolescents or adults (age 15 or older)	Share in total reported deaths, 2018-19	Excess mortality in April-May 2021 compared to the same months in the average of 2018-19 (%)
Respiratory diseases including infections other than tuberculosis	2%	24%
Heart disease/hypertension related	9%	13%
Other known chronic disease	8%	8%
Accidents/burns	2%	-63%
Causes not known	65%	137%

Table S4. HMIS-reported cause of deaths by significant categories in rural Andhra Pradesh
We chose Andhra Pradesh as the HMIS deaths in rural areas numbered 212,476 and 249,776 in 2018 and 2019, respectively. This total constituted 84% and 93% of the expected deaths based on the civil registration death totals (*4*).

		Registered/recorded deaths (000)							
**Place and population (millions)**	**Reference period and comparison months and years: pandemic vs pre-pandemic***	**During pandemic**	**During pre-pandemic**	**Excess deaths**	**Percent excess deaths in % (95% CI)**	**RGI reported coverage of CRS +**	**Actual coverage CRS based on UN death totals ++**	**Confirmed COVID deaths during reference period**	**Ratio of excess deaths to confirmed COVID deaths**
**C1**	**C2**	**C3**	**C4**	**C5=C3-C4**	**C6=(C3/C4-1)**	**C7**	**C8**	**C9**	**C10=C5/C9**
**States (Wave 1)**									
Andhra Pradesh (52.5)	Jul-Oct, 4 months (2020 vs 2018/19)	188	115	72	62.7 (55,70.4)	100%	84%	6,503	11.1
Assam (35.3)	Jul-Oct, 4 months (2020 vs 2018-19)	65	48	17	36.6 (28.7,44.5)	74%	70%	918	19.0
Tamil Nadu (82.3)	Jun-Nov, 6 months (2020 vs 2018-19)	359	282	76	27 (18.6,35.4)	100%	91%	11,545	6.6
Madhya Pradesh (84.3)	Jul-Dec, 6 months (2020 vs 2018-19)	259	232	27	11.8 (10.6,12.9)	89%	65%	3,035	0.1
Kerala, (35.3)	Aug-Dec, 5 months (2020 vs 2017-19)	182	172	10	5.6 (5,6.2)	100%	90%	4,548	2.1
Haryana (29.4)	Jul-Dec, 6 months (2020 vs 2018-19)	105	91	15	16.3 (14.9,17.7)	100%	85%	2,669	5.5
West Bengal (101)	Jul-Dec, 6 months (2020 vs 2018-19)	283	206	77	37.2 (36.4,37.9)	100%	60%	9,044	8.5
Maharashtra (125)	Jul-Dec, 6 months (2020 vs 2018-19)	321	230	91	39.7 (36.8,42.7)	100%	52%	41,666	2.2
Himachal Pradesh (7.6)	Jul-Dec, 6 months (2020 vs 2018-19)	23	20	3	14.2 (12.3,16.2)	86%	82%	913	3.1
Rajasthan, RJ (78.8)	Jul-Dec, 6 months (2020 vs 2018-19)	117	107	10	9.6 (8.8,10.5)	99%	35%	2,283	4.5
Karnataka, KN (69.5)	Jul-Dec, 6 months (2020 vs 2018-19)	327	256	70	27.4 (24.1,30.7)	100%	78%	11,844	**5.9**
	Sub-totals and medians	**2,228**	**1,759**	**469**	**27.0 (18.6,30.7)**	**100.0%**	**62.6%**	**94,968**	**5.5**
**Cities (Wave 1)**									
Ahmedabad, GJ (6.4)	April-May 2 months (2020 vs 2019)	11	5	5	95 (29.5,160.6)	100%	89%	857	6.1
Hyderabad, TL (9.7)	Jun-Dec, 7 month (2020 vs 2016-19)	50	32	18	56.7 (54.2,59.2)	97%	107%	-	-
Nagpur, MH (4.6)	July-Dec, 6 months (2020 vs 2019)	16	11	5	43.9 (29.5,58.2)	100%	92%	3,189	1.5
Mumbai, MH (18.4)	May-Nov, 7 months (2020 vs 2017-19)	75	53	22	41.3 (38.1,44.4)	100%	94%	18,209	1.2
Bangalore, KN (8.5)	Jul-Dec, 6 months (2020 vs 2019)	47	33	13	40 (36.6,43.4)	100%	128%	4,518	2.9
Chennai, TN (8.7)	Jun-Dec, 7 months (2020 vs 2015-19)	48	38	9	24.6 (23.8,25.4)	100%	112%	4,974	1.9
Kolkata, WB (14.1)	Jul-Dec, 6 months (2020 vs 2015-19)	41	35	6	17.1 (16.6,17.6)	100%	78%	5,454	1.1
	Sub-totals and medians	**287**	**208**	**79**	**41.3 (29.5,44.4)**	**100.0%**	**93.9%**	**37,201**	**1.7**
**States (Wave 2)**									
Madhya Pradesh (84.3)	Mar-May, 3 months (2021 vs 2018-19)	268	90	178	197.9 (138.6,257.2)	89%	50%	4,203	42.4
Andhra Pradesh (52.5)	Apr-Jun, 3 months (2021 vs 2018-19)	216	82	135	164.3 (111.8,216.9)	100%	80%	3,713	36.2
Tamil Nadu (82.3)	March-May, 3 months (2021 vs 2018-19)	203	140	64	45.5 (34.3,56.6)	100%	90%	11,516	5.5
Gujarat (69.5)	Mar-May, 2.3 months (2021 vs 2020)	124	86	37	43.4 (34.3,52.4)	100%	100%	5,423	6.9
Odisha (46.5)	Jan-Jun, 5.6 months (2021 vs 2015-19)	191	156	36	22.8 (17.8,27.8)	100%	96%	2,145	16.6
Kerala, (35.3)	Apr-May, 2 months (2021 vs 2017-19)	48	39	9	22.8 (16.6,28.9)	100%	82%	4,194	2.1
Haryana (29.4)	Apr-May, 2 months (2021 vs 2018-19)	74	28	46	164.7 (135.1,194.3)	100%	79%	5,148	9.0
West Bengal (101)	April-May, 2 months (2021 vs 2018-19)	117	56	61	110.6 (88,133.2)	100%	49%	5,212	11.8
Maharashtra (125)	March-May, 3 months (2021 vs 2018-19)	259	106	153	143.8 (118.5,169.1)	100%	48%	43,190	3.5
Himachal Pradesh (7.6)	April-May, 2 months (2021 vs 2018-19)	11	6	5	79.2 (52.5,106)	86%	77%	2,145	2.3
Karnataka, KN (69.5)	Apr-Jun, 3 months (2021 vs 2018-19)	217	112	105	94.1 (81.1,107.1)	100%	68%	22,473	4.7
	Sub-totals and medians	**1,729**	**901**	**829**	**94.1 (34.3,56.6)**	**100.0%**	**50.3%**	**109,362**	**6.9**
**Cities (Wave 2)**									
Hyderabad, TL (9.7)	Apr-May, 2 months (2021 vs 2016-19)	20	9	11	130.3 (127.5,133.1)	97%	103%	-	-
Bangalore, KN (8.5)	Apr-May, 2 months (2021 vs 2019)	22	11	12	110.2 (69.4,150.9)	100%	124%	9,723	1.2
Chennai, TN (8.7)	Mar-Apr, 2 months (2021 vs 2018-19)	12	10	2	17.9 (15.6,20.2)	100%	102%	773	2.3
Mumbai, MH (18.4)	Mar-May, 3 months (2020 vs 2017-19)	30	21	9	42.5 (32.4,52.5)	100%	88%	3,480	2.6
Kolkata, WB (14.1)	Mar-Apr, 2 months (2021 vs 2018-19)	20	10	10	94.4 (81.6,107.2)	100%	69%	663	14.7
	Sub-totals and medians	**105**	**61**	**44**	**94.4 (69.4,107.2)**	**100.0%**	**101.9%**	**14,639**	**2.5**

Notes: CRS=Civil Registration System. * The state-wise pandemic and pre-pandemic CRS deaths in columns C3 and C4 are from the recent government data (see Table S6 for primary data). + The Registrar General of India 2018 report on coverage of CRS deaths by state (*13*). ++ Pre-pandemic deaths in the CRS divided by the estimated UN death totals (*20*), which we partitioned to state-level totals using the Sample Registration System death rates (*13*).

Table S5. Excess deaths compared to CRS baseline rates, completeness of death registration in Indian states and confirmed COVID deaths and ratio of excess to confirmed by pandemic waves for states and cities of India

State/City/ Month	Year / No. of deaths 2015	2016	2017	2018	2019	2020	2021
**India States**							
Andhra Pradesh State (GitHub-DDL, GitHub-local mortality)							
1				33,637	32,302	31,989	37,963
2				28,788	31,170	26,802	31,920
3				27,341	27,972	25,413	33,367
4				25,979	26,290	22,362	39,102
5				24,357	29,928	28,066	134,041
6				27,006	30,201	28,440	43,296
7				26,458	28,465	34,757	
8				27,929	28,667	54,940	
9				26,626	30,447	55,874	
10				28,478	33,631	42,104	
11				26,785	33,201	38,665	
12				29,891	31,140	39,495	
Assam State (GitHub-local mortality)							
1				12,204	15,680	18,556	
2				12,193	14,806	17,971	
3				12,155	14,266	16,975	
4				11,826	13,147	9,003	
5				11,761	12,169	8,655	
6				11,656	11,303	8,305	
7				11,456	11,065	9,118	
8				11,378	10,925	16,887	
9				11,593	13,177	18,364	
10				11,755	14,117	20,814	
11				11,515	15,700	21,780	
12				13,113	16,702	20,657	
Madhya Pradesh State (GitHub-local mortality)							
1				34,451	39,528	41,281	44,133
2				29,986	36,005	34,645	36,535
3				28,504	32,911	29,747	34,667
4				26,949	28,599	24,198	68,535
5				32,456	30,506	34,320	164,838
6				33,818	38,978	37,399	
7				36,523	38,922	41,303	
8				38,230	44,151	40,213	
9				40,180	43,517	47,315	
10				37,766	37,953	45,208	
11				30,349	39,124	40,283	
12				37,960	39,625	45,145	
Tamil Nadu State (GitHub-local mortality)							
1				55,390	58,405	58,132	62,273
2				47,231	46,560	48,893	52,845
3				46,703	47,772	45,987	50,959
4				43,586	45,913	41,482	58,775
5				43,928	51,639	50,834	93,573
6				43,222	48,868	50,687	
7				42,633	45,762	60,052	
8				41,051	47,385	70,033	
9				40,484	48,621	65,447	
10				46,337	53,028	60,096	
11				51,602	55,658	52,231	
12				34,025	38,610	40,417	
Kerala state (GitHub-DDL)							
1			20,326	22,081	23,676	21,357	23,328
2			18,128	19,354	19,892	19,714	21,152
3			19,013	19,843	20,640	19,581	20,706
4			19,014	18,825	19,983	17,212	20,229
5			20,053	19,370	20,514	17,680	27,957
6			22,597	20,435	20,509	19,346	5,908
7			26,302	23,372	24,567	20,963	.
8			23,101	25,683	25,475	23,394	.
9			21,513	22,844	24,318	22,704	.
10			20,681	21,075	22,005	24,949	.
11			20,083	21,031	21,626	22,115	.

12	21,292	21,681	20,945	23,406	.
Gujarat state (Media published Death Certificate issues and CR data)					
3					26,026
4			86,410		57,796
5*			CR Report		40,051
Haryana state					
1		17,818	17,638	19,066	17,858
2		14,614	15,268	15,727	14,908
3		14,567	15,316	14,787	14,908
4		13,580	13,658	12,965	28,276
5		14,335	14,630	15,445	46,108
6		14,772	14,946	15,496	
7		13,115	13,486	15,590	
8		13,648	14,871	15,581	
9		14,079	14,861	17,253	
10		15,564	15,003	16,611	
11		15,941	15,527	20,914	
12		16,601	18,591	19,468	
Himachal Pradesh State					
1		4,369	3,990	4,089	3,878
2		3,598	3,794	3,542	3,215
3		3,507	3,701	3,175	3,051
4		3,013	3,009	2,904	3,769
5		3,272	3,021	3,096	7,267
6		3,048	3,285	3,053	.
7		2,932	2,945	2,982	.
8		2,988	3,176	3,278	.
9		3,042	3,174	3,530	.
10		3,315	3,330	3,665	.
11		3,255	3,495	4,647	.
12		3,794	4,050	4,455	.
West Bengal State					
1		41,157	41,552	49,345	49,023
2		30,017	34,830	41,848	40,148
3		28,181	34,746	34,263	33,739
4		23,880	29,984	31,244	44,822
5		25,977	31,196	34,493	72,085
6		25,842	33,396	35,463	2,128
7		25,919	34,759	41,786	.
8		27,968	33,809	45,453	.
9		29,505	31,456	43,876	.
10		31,319	37,651	46,473	.
11		32,462	39,559	50,691	.
12		39,103	48,593	54,390	.
Maharashtra State					
1		40,214	41,092	42,448	41,360
2		34,945	34,139	37,213	37,391
3		34,373	36,951	35,077	52,197
4		32,105	35,865	34,328	117,723
5		35,493	37,957	48,610	89,452
6		30,526	35,405	46,496	.
7		34,736	37,508	52,137	.
8		35,996	42,983	60,220	.
9		38,162	41,847	66,453	.
10		37,942	40,028	52,260	.
11		34,632	38,191	45,077	.
12		37,365	40,062	44,866	
Rajasthan (GitHub-DDL)					
1		20,798	20,239	21,954	19,622
2		18,301	18,089	18,056	14,860
3		18,921	17,784	16,378	16,084
4		18,018	16,651	17,596	26,251
5		19,446	17,603	20,582	49,044
6		17,048	19,766	17,959	
7		15,640	15,656	17,151	
8		16,023	17,569	17,918	
9		15,812	19,291	18,719	
10		17,226	17,924	18,405	
11		17,489	17,794	21,265	
12		21,648	21,448	23,581	

India Cities

Ahmedabad City (Newspaper reporting)							
4					2,784	3,558	
5					2,706	7,150	
Bangalore (Urban) (GitHub-DDL)							
1			.	.	5,168	5,983	6,012
2			.	.	5,766	5,454	5,538
3			.	.	5,400	4,716	6,216
4			.	.	4,806	3,327	6,249
5			.	.	5,899	4,469	16,249
6			.	.	4,687	4,881	.
7			.	.	5,278	6,477	.
8			.	.	5,481	9,340	.
9			.	.	5,411	8,710	.
10			.	.	5,597	8,413	.
11			.	.	5,982	7,342	.
12			.	.	5,544	6,329	.
Chennai City (GitHub-local mortality)							
1	5,543	5,644	5,833	5,837	6,228	5,915	6,195
2	4,753	4,528	4,877	5,132	5,114	5,046	5,218
3	4,959	4,633	4,925	5,107	5,286	4,961	6,038
4	4,505	4,611	4,735	4,772	4,964	4,149	11,876
5	5,072	5,102	8,142	5,214	5,758	6,650	5,815
6	4,698	4,690	4,890	5,065	6,071	7,747	-
7	4,499	4,717	5,156	5,003	5,121	6,368	-
8	4,484	4,712	5,259	4,814	5,351	6,805	-
9	4,460	4,609	5,728	4,828	5,457	7,087	-
10	5,019	4,572	5,603	5,762	6,147	6,714	-
11	6,064	4,756	5,470	5,770	5,978	6,115	-
12	5,842	4,610	4,691	4,802	4,885	6,440	-
Hyderabad (GitHub-local mortality)							
1		4,033	4,455	4,841	5,470	5,763	5,879
2		3,319	3,613	4,354	4,628	5,199	4,749
3		3,664	4,035	4,277	4,851	4,894	5,090
4		3,979	4,016	4,002	4,708	3,967	9,465
5		3,844	4,526	4,222	6,000	5,061	10,858
6		3,567	3,669	3,746	4,707	7,011	
7		4,152	3,954	4,103	4,808	10,423	
8		4,525	4,220	4,271	5,620	7,974	
9		3,961	4,525	4,615	6,048	7,013	
10		4,255	4,650	4,925	6,117	6,212	
11		4,087	4,427	4,700	5,466	5,708	
12		4,121	4,603	4,952	5,689	5,996	
Kolkata City (GitHub-local mortality)							
1	6,441	6,598	7,103	8,506	7,918	7,587	6,293
2	5,598	4,889	5,646	5,934	6,421	6,115	4,776
3	5,279	4,923	5,434	5,346	6,078	5,377	4,374
4	4,582	4,978	5,246	4,861	5,128	4,830	10,256.29
5	4,890	4,669	5,476	4,904	5,314	5,375	6,615.86
6	4,421	4,783	4,730	5,066	5,395	5,007	-
7	4,754	5,391	5,549	4,915	5,329	6,590	-
8	5,274	5,610	6,275	5,532	5,056	6,521	-
9	5,389	5,257	5,704	5,612	5,092	6,050	-
10	5,194	5,619	5,905	5,804	5,836	6,986	-
11	5,170	5,924	6,337	5,685	5,805	7,238	-
12	5,739	5,665	5,747	6,012	5,502	7,096	-
Nagpur City (GitHub-local mortality)							
3						1,415	3,168
4					1,583	1,311	
5					1,900	1,624	
6					1,678	1,512	
7					1,590	1,808	
8					1,787	3,385	
9					1,958	4,096	
10					1,801	2,387	
11					1,775	2,325	
12					2,166	1,934	
Mumbai City (GitHub-local mortality)							
1			8,004	8,306	8,324	8,397	7,732
2			7,158	7,305	7,797	7,116	7,131

3	7,810	7,436	7,155	5,703	8,302
4	6,234	6,719	6,752	5,537	14,484
5	6,960	7,407	7,335	9,161	7,514
6	7,068	6,874	6,732	15,756	.
7	7,675	7,336	7,931	11,770	.
8	7,247	7,372	8,164	10,215	.
9	8,250	7,231	7,953	10,061	.
10	7,700	8,755	7,390	9,835	.
11	7,943	7,235	8,320	8,013	.
12	6,988	6,876	7,370	7,834	
**National**					
(HMIS, Ministry of Health and Family Welfare, GoI)					
1		221,543	238,079	244,345	234,907
2		204,119	209,475	211,623	212,211
3		192,123	198,159	183,436	203,316
4		167,999	186,274	164,831	311,688
5		188,632	206,479	186,395	513,386
6		191,197	211,723	191,544	
7		191,412	207,594	212,556	
8		197,917	226,277	250,096	
9		210,200	232,270	270,371	
10		214,351	224,700	246,526	
11		213,179	225,872	247,578	
12		215,885	230,308	247,870	
**Rural**					
1		208,822	224,275	228,066	198,225
2		189,933	197,359	197,156	178,885
3		179,776	186,503	171,650	170,873
4		156,552	172,858	139,978	261,854
5		177,211	192,592	156,955	434,082
6		180,094	197,500	159,028	
7		179,171	193,672	177,083	
8		184,759	211,131	209,647	
9		198,113	217,423	225,991	
10		201,454	209,953	205,698	
11		200,740	210,914	207,145	
12		203,293	214,627	209,394	
**Urban**					
1		12,721	13,804	16,279	21,682
2		14,186	12,116	14,467	18,326
3		12,347	11,656	11,786	17,443
4		11,447	13,416	9,847	34,831
5		11,421	13,887	14,435	64,292
6		11,103	14,223	17,516	
7		12,241	13,922	20,471	
8		13,158	15,146	25,443	
9		12,087	14,847	29,380	
10		12,897	14,747	25,828	
11		12,439	14,958	25,433	
12		12,592	15,681	23,471	

Table S6. Input data used to create Table 1

Data from the Development Data Lab (DDL) are available at https://github.com/devdatalab/covid (doi:10.5281/zenodo.5796813) (*34*).

References and Notes

1. "The Coronavirus App" (Scriby, Inc, 2021); https://coronavirus.app/map.

2. COVID-19 India Data Operations Group, "Covid19India.org" (2021); https://www.covid19india.org/.

3. G. R. Menon, L. Singh, P. Sharma, P. Yadav, S. Sharma, S. Kalaskar, H. Singh, S. Adinarayanan, V. Joshua, V. Kulothungan, J. Yadav, L. K. Watson, S. A. Fadel, W. Suraweera, M. V. V. Rao, R. S. Dhaliwal, R. Begum, P. Sati, D. T. Jamison, P. Jha, National Burden Estimates of healthy life lost in India, 2017: An analysis using direct mortality data and indirect disability data. *Lancet Glob. Health* **7**, e1675–e1684 (2019). doi:10.1016/S2214-109X(19)30451-6 Medline

4. Office of the Registrar General, India, "Vital Statistics of India based on the Civil Registration System 2019" (2021); https://censusindia.gov.in/2011-Common/CRS_2019/CRS2019_report.pdf.

5. C. Z. Guilmoto, Estimating the death toll of the Covid-19 pandemic in India. medRxiv 2021.06.29.21257965 [Preprint]. 2 July 2021. https://www.medrxiv.org/content/10.1101/2021.06.29.21257965v1.

6. C. T. Leffler, J. D. Lykins V, E. Yang, Preliminary Analysis of Excess Mortality in India During the Covid-19 Pandemic. medRxiv 2021.08.04.21261604 [Preprint]. 27 September 2021. https://www.medrxiv.org/content/10.1101/2021.08.04.21261604v2.

7. A. Malani, S. Ramachandran, "Using Household Rosters from Survey Data to Estimate All-cause Mortality during COVID in India" (2021); https://www.nber.org/papers/w29192.

8. M. Banaji, A. Gupta, Estimates of pandemic excess mortality in India based on civil registration data. medRxiv 2021.09.30.21264376 [Preprint]. 1 October 2021. https://www.medrxiv.org/content/10.1101/2021.09.30.21264376v1.

9. A. Gupta, M. Banaji, "Lessons from India's all-cause mortality data," *The Hindu* (2021); https://www.thehindu.com/opinion/lead/lessons-from-indias-all-cause-mortality-data/article36007188.ece.

10. A. Anand, J. Sandefur, A. Subramanian, "Three New Estimates of India's All-Cause Excess Mortality during the COVID-19 Pandemic" (2021); https://www.cgdev.org/publication/three-new-estimates-indias-all-cause-excess-mortality-during-covid-19-pandemic.

11. R. J. Acosta *et al.*, All-cause excess mortality in the State of Gujarat, India, during the COVID-19 pandemic. medRxiv 2021.08.22.21262432 [Preprint]. 25 August 2021. https://www.medrxiv.org/content/10.1101/2021.08.22.21262432v1.

12. A. Bamezai *et al.*, Survey evidence of excess mortality in Bihar in the second COVID-19 surge. SocArXiv [Preprint]. 29 July 2021. https://osf.io/preprints/socarxiv/zxq97/.

13. Office of the Registrar General & Census Commissioner, India, "SRS statistical report 2018" (2020); https://censusindia.gov.in/vital_statistics/SRS_Reports_2018.html.

14. P. E. Brown *et al.*, Mortality from COVID in Colombia and Peru: Analyses of Mortality Data and Statistical Forecasts. medRxiv 2020.08.24.20181016 [Preprint]. 16 November 2020. https://www.medrxiv.org/content/10.1101/2020.08.24.20181016v2.

15. World Health Organization, “The true death toll of COVID-19: estimating global excess mortality”; https://www.who.int/data/stories/the-true-death-toll-of-covid-19-estimating-global-excess-mortality.

16. R. S, “Deaths By 'Unknown Causes' On National Health Mission Portal 2X Official Covid Toll,” *IndiaSpend* (2021); https://www.indiaspend.com/covid-19/deaths-unknown-causes-national-health-mission-portal-covid-toll-760219.

17. A. Velumani *et al.*, SARS-CoV-2 Seroprevalence in 12 Cities of India from July-December 2020. medRxiv 2021.03.19.21253429 [Preprint]. 24 March 2021. https://www.medrxiv.org/content/10.1101/2021.03.19.21253429v1.

18. Team CVoter, “COVID-19 Tracker Surveys in India” (2020); https://cvoterindia.com/wp-content/uploads/2020/Covid_Tracker_Methodology_Note.pdf.

19. Team CVoter, “Cvoter News Services”; https://cvoterindia.com/trackers/.

20. United Nations, Department of Economic and Social Affairs, Population Division, “World Population Prospects 2019: Highlights” (2019); https://population.un.org/wpp/Publications/Files/WPP2019_Highlights.pdf.

21. Population Reference Bureau, “International Indicators: Average Household Size” (2020); https://www.prb.org/international/indicator/hh-size-av/map/country.

22. S. H. Woolf, D. A. Chapman, R. T. Sabo, E. B. Zimmerman, Excess Deaths From COVID-19 and Other Causes in the US, March 1, 2020, to January 2, 2021. *JAMA* **325**, 1786–1789 (2021). doi:10.1001/jama.2021.5199 Medline

23. Ministry of Health and Family Welfare, Government of India, “Health Management Information System” (2021); https://hmis.nhp.gov.in/#!/standardReports.

24. M. R. Nepomuceno *et al.*, Sensitivity of excess mortality due to the COVID-19 pandemic to the choice of the mortality index, method, reference period, and the time unit of the death series. medRxiv 2021.07.20.21260869 [Preprint]. 23 July 2021. https://www.medrxiv.org/content/10.1101/2021.07.20.21260869v1.

25. P. Jha, Reliable direct measurement of causes of death in low- and middle-income countries. *BMC Med.* **12**, 19 (2014). doi:10.1186/1741-7015-12-19 Medline

26. C. Pattabiraman, P. Prasad, A. K. George, D. Sreenivas, R. Rasheed, N. V. K. Reddy, A. Desai, R. Vasanthapuram, Importation, circulation, and emergence of variants of SARS-CoV-2 in the South Indian state of Karnataka. *Wellcome Open Res.* **6**, 110 (2021). doi:10.12688/wellcomeopenres.16768.1

27. C. Meh, A. Sharma, U. Ram, S. Fadel, N. Correa, J. W. Snelgrove, P. Shah, R. Begum, M. Shah, T. Hana, S. H. Fu, L. Raveendran, B. Mishra, P. Jha, Trends in maternal mortality in India over two decades in nationally representative surveys. *BJOG* 1471-0528.16888 (2021). doi:10.1111/1471-0528.16888 Medline

28. P. Madahar, H. Wunsch, P. Jha, A. S. Slutsky, D. Brodie, Trends in COVID-19-related in-hospital mortality: Lessons learned from nationwide samples. *Lancet Respir. Med.* **9**, 322–324 (2021). doi:10.1016/S2213-2600(21)00080-1 Medline

29. Centre for Global Health Research, cghr-toronto/Indian_Covid_Mortality: Indian Covid Mortality, Zenodo (2021). doi.org/10.5281/zenodo.5796647. https://github.com/cghr-toronto/Indian_Covid_Mortality.

30. Reuters Staff, "Table-India's mobile subscriber base rises in Jan; Airtel adds 5.9 mln (March 17)," *Reuters* (2021); https://www.reuters.com/article/india-telecoms-users-idUKL4N2LF2GQ.

31. S. Keeter, "From Telephone to the Web: The Challenge of Mode of Interview Effects in Public Opinion Polls" (Pew Research Center, 2015); https://www.pewresearch.org/methods/2015/05/13/from-telephone-to-the-web-the-challenge-of-mode-of-interview-effects-in-public-opinion-polls/

32. P. Gerland, UN Population Division's Methodology in Preparing Base Population for Projections: Case study for India. *Asian Popul. Stud.* **10**, 274–303 (2014). doi:10.1080/17441730.2014.947059

33. Office of the Registrar General, India, "Report on medical certification of cause of death 2019" (2021); https://censusindia.gov.in/2011-Documents/mccd_Report1/MCCD_Report_2019.pdf.

34. P. Novosad *et al.*, devdatalab/covid: DDL COVID repo, Zenodo (2021). doi.org/10.5281/zenodo.5796813. https://github.com/devdatalab/covid.

35. J. A. Lewnard, A. Mahmud, T. Narayan, B. Wahl, T. S. Selvavinayagam, C. Mohan B, R. Laxminarayan, All-cause mortality during the COVID-19 pandemic in Chennai, India: An observational study. *Lancet Infect. Dis.* S1473-3099(21)00746-5 (2021). doi:10.1016/S1473-3099(21)00746-5 Medline
